# 54. The Effect of Targeting High-Risk Patients for Antimicrobial Stewardship Intervention on Hospital-Onset *Clostridioides Difficile* Infection Rates

**DOI:** 10.1093/ofid/ofab466.256

**Published:** 2021-12-04

**Authors:** Albert Yang, Monica Donnelley

**Affiliations:** 1 University of California, Davis Health, Sacramento, California; 2 UC Davis Medical Center, Davis, California

## Abstract

**Background:**

*Clostridioides difficile* infection contributes to significant burden on patients and the healthcare system, costing billions in excess costs every year for hospital care. Continued use of antibiotics after *C. difficile* infection diagnosis is a risk factor for recurrent infection. Also, individuals who have had a recurrence of *C. difficile* infection are at a higher risk of subsequent episodes.

**Methods:**

This prospective, observational, pre-post study evaluated the effect of implementing a targeted antimicrobial stewardship initiative towards a high-risk target population on the rate of in-hospital C. difficile infection rates. High-risk targets were identified through an electronic health system report of admitted patients at a large academic medical center who were toxin assay positive or had a documented history of *C. difficile* infection. Subjects who met the criteria were assessed for interventions by the pharmacy-driven antimicrobial stewardship service. The primary outcome compared the hospital-onset *C. difficile* rates and standardized infection ratio (SIR) before and after implementation of the initiative. The SIR is reported to the National Healthcare Safety Network (NHSN) and is calculated as a ratio between the number of observed and predicted infections, which is adjusted for facility-specific factors that contribute *C. difficile* risk. Negative binomial regression was used to calculate the predicted *C. difficile* infections in the SIR. Poisson regression was used to generate a 95% prediction interval for the predicted C. difficile infection rate.

**Results:**

The mean age of subjects was 63 years old and 85% had no history of prior *C. difficile* infection. The most common intervention was de-escalation of antibiotics (46%). The post-implementation SIR was 0.55 and hospital-onset *C. difficile* rate was 13, both of which were significantly lower than predicted.

**Conclusion:**

Targeting patients who have a history of or are newly diagnosed with *C. difficile* infection may decrease hospital-onset *C. difficile* rates.

**Disclosures:**

**All Authors**: No reported disclosures

